# Appraising the quality of guidelines for caries management using AGREE II: a systematic review

**DOI:** 10.1093/eurpub/ckac131.364

**Published:** 2022-10-25

**Authors:** D Lamloum, P Ferrara, A Arghittu, P Castiglia, M Gaeta, A Odone, G Campus

**Affiliations:** 1 Department of Public Health, University of Pavia, Pavia, Italy; 2 Department of Dentistry, University of Sassari, Sassari, Italy; 3 Department of Dentistry, University of Bern, Bern, Switzerland

## Abstract

**Background:**

Caries is one among the most prevalent dental disease and its prevention and treatment are crucial from both dental care and public health perspectives. Yet, caries’ management greatly varies across contexts according to the availability of specific Clinical Practice Guidelines (CPGs). Here, we present the results of a systematic review aimed at the appraisal of the current available CPGs on caries prevention and treatment.

**Methods:**

A literature search was performed in PubMed, EMBASE, SCOPUS, and seven relevant guidelines databases up to March 2022, exploring CPGs published from 2012. The literature review was conducted in accordance with PRISMA guidelines. The Appraisal of Guidelines, Research and Evaluation (AGREE) II checklist was used to measure the methodological rigour and quality of the retrieved CPGs.

**Results:**

The systematic search resulted in a total of 1403 records, and 21 CPGs met the inclusion criteria. Overall, these considered different aspects of caries prevention and treatment. Regarding the appraisal through the AGREE II tool, the overall median score was 60.2% and 11 out of 21 CPGs were classified as “Recommended”, while the others as “Recommended with modification”. The domain analysis showed that the highest median scores were reached for Scope and Purpose (88.9%), Clarity of Presentation (86.9%), and Rigor of Development (67.8%), while the lowest were seen for Stakeholder Involvement (63.3%), Applicability (17.5%), and Editorial Independence (50%).

**Conclusions:**

This systematic review showed that the rigor of CPGs for caries prevention and treatment remained suboptimal according to AGREE II evaluation, and highlighted that more efforts are needed to improve their quality. The AGREE II checklist is a comprehensive and easy-to-use tool for the development of CPGs, and its use ensures that evidence-based approaches are incorporated into consistent recommendations for the translation of evidence into practice.

**Key messages:**

• The rigor of CPGs for caries prevention and treatment is suboptimal according to AGREE II evaluation, however, more efforts are needed to improve their quality.

• The AGREE II checklist is a comprehensive and easy-to-use tool for the development of CPGs.

